# Patient and physician discordance of global disease assessment in juvenile dermatomyositis: findings from the Childhood Arthritis & Rheumatology Research Alliance Legacy Registry

**DOI:** 10.1186/s12969-020-0402-x

**Published:** 2020-01-15

**Authors:** Heather Tory, David Zurakowski, Susan Kim, the CARRA Juvenile Dermatomyositis Quality Measures Workgroup for the CARRA Registry Investigators, L. Abramson, E. Anderson, M. Andrew, N. Battle, M. Becker, H. Benham, T. Beukelman, J. Birmingham, P. Blier, A. Brown, H. Brunner, A. Cabrera, D. Canter, D. Carlton, B. Caruso, L. Ceracchio, E. Chalom, J. Chang, P. Charpentier, K. Clark, J. Dean, F. Dedeoglu, B. Feldman, P. Ferguson, M. Fox, K. Francis, M. Gervasini, D. Goldsmith, G. Gorton, B. Gottlieb, T. Graham, T. Griffin, H. Grosbein, S. Guppy, H. Haftel, D. Helfrich, G. Higgins, A. Hillard, J. R. Hollister, J. Hsu, A. Hudgins, C. Hung, A. Huttenlocher, N. Ilowite, A. Imlay, L. Imundo, C. J. Inman, J. Jaqith, R. Jerath, L. Jung, P. Kahn, A. Kapedani, D. Kingsbury, K. Klein, M. Klein-Gitelman, A. Kunkel, S. Lapidus, S. Layburn, T. Lehman, C. Lindsley, M. Macgregor-Hannah, M. Malloy, C. Mawhorter, D. McCurdy, K. Mims, N. Moorthy, D. Morus, E. Muscal, M. Natter, J. Olson, K. O’Neil, K. Onel, M. Orlando, J. Palmquist, M. Phillips, L. Ponder, S. Prahalad, M. Punaro, D. Puplava, S. Quinn, A. Quintero, C. Rabinovich, A. Reed, C. Reed, S. Ringold, M. Riordan, S. Roberson, A. Robinson, J. Rossette, D. Rothman, D. Russo, N. Ruth, K. Schikler, A. Sestak, B. Shaham, Y. Sherman, M. Simmons, N. Singer, S. Spalding, H. Stapp, R. Syed, E. Thomas, K. Torok, D. Trejo, J. Tress, W. Upton, R. Vehe, E. von Scheven, L. Walters, J. Weiss, P. Weiss, N. Welnick, A. White, J. Woo, J. Wootton, A. Yalcindag, C. Zapp, L. Zemel, A. Zhu

**Affiliations:** 1 Division of Pediatric Rheumatology, Connecticut Children’s Medical Center, 282 Washington Street, Hartford, CT 06106 USA; 2 Department of Pediatrics, University of Connecticut School of Medicine, Farmington, CT USA; 3 Departments of Anesthesiology and Surgery, Boston Children’s Hospital, Harvard Medical School, Boston, MA USA; 4 Pediatric Rheumatology, Benioff Children’s Hospital and University of San Francisco Medical Center, San Francisco, CA USA

**Keywords:** Patient reported outcome measures, Juvenile Dermatomyositis, Quality of life, Physician perspective, Patient perspective

## Abstract

**Background:**

Global disease activity scores (gVAS) capture patient or family (PF) and physician (MD) assessments of disease. This study sought to measure discordance between PF and MD global activity scores in juvenile dermatomyositis (JDM), and determine factors associated with discordance.

**Methods:**

Patients with JDM were included from the Childhood Arthritis and Rheumatology Research Alliance (CARRA) Legacy Registry (*N* = 563). PF and MD gVAS were assessed for discordance, defined as a ≥ 2-point difference. Factors associated with discordant gVAS were compared in univariate analysis. Multivariable regression analysis was used to identify predictors of discordance.

**Results:**

Almost 40% (*N* = 219) of PF and MD gVAS were discordant. Among discordant scores, 68% of PF rated gVAS ≥2-points above MD, which was associated with calcinosis and lower quality of life and functional scores (*p* < 0.01). MD gVAS rated ≥2-points above PF in 32%, which was associated with abnormal laboratory results, weakness, arthritis, rash and other skin changes, and current intravenous steroid treatment (*p* < 0.01). In multivariate analysis, predictors for higher PF rating included calcinosis, lower quality of life and functional scores, while predictors for higher MD rating included rash, calcinosis, nailfold capillaroscopy changes, and current intravenous steroid treatment.

**Conclusions:**

Discordance between PF and MD gVAS was common in this JDM cohort. Overall, higher PF rating was associated with poorer patient reported outcome (PRO) scores, while higher MD rating was associated with poorer objective measures. This suggests PF and MD assessments of gVAS may be measuring different aspects of disease, highlighting the importance of integrating PROs into clinical practice and research.

## Background

Juvenile dermatomyositis (JDM) is the most common chronic inflammatory myopathy of childhood [1]. It is a systemic vasculopathy characterized by pathognonomic rashes and proximal muscle weakness. Accurate assessment of disease activity is essential in directing medical care; however, since no single biomarker of disease activity exists for JDM, healthcare providers use a combination of clinical, laboratory and diagnostic measures for assessment. Patient reported outcome measures (PROs) in JDM are not typically monitored in standard clinical practice, despite evidence of the importance of integrating the patient perspective in assessment of disease status [2].

Standardized disease activity measures have been developed by the International Myositis Assessment and Clinical Studies Group (IMACS) and the Pediatric International Trials Organisation (PRINTO), and are recommended for use in all myositis therapeutic trials and clinical studies [3]. Both the IMACS and PRINTO Disease Activity Core Sets Measures include some PROs, including the Patient/Parent Global Activity Assessment Score (PF gVAS), as well as the Physician Global Activity Assessment Score (MD gVAS).

The PF gVAS and MD gVAS are partially validated tools, meant to measure the global evaluation of overall disease activity using a 10 cm visual analog scale (VAS), where “0”represents no disease activity and “10” represents severe disease activity [4]. These measures are also included in the recently accepted Myositis Response Criteria for JDM, which were developed to define minimal, moderate and major clinical response to treatments in both adult and pediatric myositis, and are recommended for use as primary endpoints in myositis therapeutic trials [5].

Discordance between patient and physician global assessments of disease activity has been reported in several rheumatologic conditions, including rheumatoid arthritis and psoriatic arthritis [6, 7]. Discordance has also been reported between patients/families and physicians in physical function measures in juvenile idiopathic arthritis, with poorer patient/family scores seen in association with poorer scores on PROs, and poorer physician scores associated with poorer objective markers [8]. However, to our knowledge, discordance between patient/family and physician global assessments has not been previously reported in JDM.

In this study, we compared patient/family and physician global assessments of disease activity in JDM and sought to identify predictors of discordance.

## Methods

### Setting and study population

The study population included a cross-sectional cohort of patients with physician-diagnosed JDM enrolled in the North American multi-center Childhood Arthritis and Rheumatology Research Alliance (CARRA) Legacy Registry (CLR) over a 5-year period (2010–2015). A subset of patients with JDM enrolled in this registry has been previously described [9].

Data was abstracted from the baseline enrollment visit using a standardized form. Data included demographics, medication history, PRO measures (global disease activity assessment on a 10 point visual analog scale (VAS), Childhood Health Assessment Questionnaire (CHAQ), overall pain score using a 10 point VAS or the Faces Pain Scale based on age, and overall quality of life scores using a 5 point Likert scale of very poor to excellent) as reported by the patient or the family member (it was not possible to confirm attribution), and physician reported outcome measures (global activity assessment on a 10 point VAS, proximal muscle weakness using a 4 point Likert scale of none, mild, moderate or severe, muscle strength scoring using the Childhood Myositis Assessment Scale (CMAS), muscle enzyme testing, examination findings and associated co-morbidities). Global disease activity scores for patients/families and physicians were assessed by asking respondents to rate disease activity over the past week using a 10 point VAS scale.

Patients were in various stages of disease duration and severity at the time of enrollment. Those with incomplete data for the variables of physician and patient global activity assessment scores were excluded.

### Definition of discordance in global activity assessment scores

Our primary outcome was patient/parent and physician global activity assessment scores, based on a standard 10-point VAS. There is no standardization regarding definitions for discordant or concordant scores when comparing global activity assessment scores between parents/patients and physicians [6]. Based on prior studies, we defined a greater than or equal to 2 point difference between a PF gVAS and MD gVAS as discordant [10, 11]. PF gVAS and MD gVAS scores within 2 points were defined as concordant. Based on these definitions, discordant scores could have PF gVAS greater than MD gVAS (meaning the patient/parent rated the patient as having more disease activity compared to the physician) or could have MD gVAS greater than PF gVAS (meaning the physician rated the patient as having more disease activity compared to the patient/parent).

### Statistical analysis

We assessed discordance between PF gVAS and MD gVAS, defined as at least a 2 point difference, and then evaluated factors associated with this discordance for each of two possible discordant scenarios: PF > MD and MD > PF. For univariate associations, chi-square was used to compare categorical variables. Multivariate logistic regression analysis was applied to identify variable that were independent predictors of discordance with adjusted odds ratios and 95% confidence intervals as displayed in a forest plot figure. Two-tailed values of *p* < 0.05 were considered statistically significant. Statistical analysis was performed using the IBM SPSS software package (version 24.0, IBM Corporation, Armonk, NY).

## Results

### Demographics and clinical characteristics

From 2010 to 2014, 639 JDM patients were enrolled to the CLR, of which 563 patients had PF and MD gVAS data available for analysis. Mean age at enrollment was 10.6 years (range 6.9–14.7) with average age of onset 5.5 years (3.6–9.3). Most patients were female (*n* = 403, 72%), Caucasian (*n* = 442, 79%) and non-Hispanic (*n* = 471, 84%) (Table 1).

**Table 1:** Demographic information for the patients with JDM in the CARRA Legacy Registry

		Comparison of Global Assessment Scores		
Variable	Overall Cohort (*N* = 563)	Concordant gVAS: (*N* = 344)	Discordant gVAS: PF gVAS > MD gVAS (*N* = 149)	Discordant gVAS: MD gVAS > PF gVAS (*N* = 70)
Current Age, years Ϯ	10.6 (6.9–14.7)	10.8 (6.9–14.7)	10.2 (6.7–14.2)	10.4 (7.8–14.8)
Age at Onset, years Ϯ	5.5 (3.6–9.3)	5.5 (3.6–9.2)	5.3 (3.6–9.2)	5.8 (3.6–10.0)
Disease Duration, years Ϯ	3.1 (1.3–6.4)	3.4 (1.7–6.0)	2.9 (1.3–6.8)	2.8 (1.0–7.0)
Gender, no. (%)				
Male	160 (28)	99 (29)	43 (29)	18 (26)
Female	403 (72)	245 (71)	106 (71)	52 (74)
Race, no. (%)				
Caucasian	442 (79)	280 (81)	110 (74)	52 (74)
Black	68 (12)	38 (11)	20 (13)	10 (14)
Asian	20 (4)	11 (3)	7 (5)	2 (3)
Other	33 (6)	15 (5)	12 (8)	6 (9)
Ethnicity, no. (%)				
Non-Hispanic	471 (84)	287 (83)	126 (85)	58 (83)
Hispanic	92 (16)	7 (17)	23 (15)	12 (17)
Income Level, no. (%)				
< $50,000	162 (36)	90 (33)	53 (44)	19 (34)
> $50,000	283 (64)	180 (67)	66 (56)	37 (66)
CMAS, no. (%)				
< 48	122 (37)	74 (35)	29 (38)	19 (44)
> 48	211 (63)	139 (65)	48 (62)	24 (56)
Weakness, no. (%) ^a^				
None or Mild	494 (89)	311 (92)	130 (90)	53 (76)
Moderate or Severe	60 (11)	28 (8)	15 (10)	17 (24)
Muscle Enzyme, no. (%) ^a^				
Abnormal	97 (18)	48 (15)	24 (17)	25 (36)
Normal	446 (82)	284 (85)	117 (83)	45 (64)
JDM Rash, no. (%) ^a^				
Yes	289 (52)	160 (47)	68 (47)	61 (87)
No	265 (48)	180 (53)	76 (53)	9 (13)
Nailfold Changes, no. (%) ^a^				
Yes	227 (42)	126 (38)	52 (37)	49 (72)
No	313 (58)	206 (62)	88 (63)	19 (28)
Calcinosis, no. (%) ^a^				
Yes	53 (10)	18 (5)	21 (15)	14 (20)
No	487 (90)	310 (95)	121 (85)	56 (80)
GI-Cardiac, no. (%)				
Yes	17 (3)	6 (2)	7 (5)	4 (6)
No	546 (97)	338 (98)	142 (95)	66 (94)
Joint Involvement, no. (%) ^a^				
Yes	72 (13)	34 (10)	20 (14)	18 (36)
No	481 (87)	306 (90)	124 (86)	51 (64)
Pulse Steroids Use, no. (%) ^a^				
Yes	69 (14)	32 (10)	20 (15)	17 (27)
No	441 (86)	283 (90)	113 (85)	45 (73)
Oral Steroids Use, no. (%) ^a^				
Yes	261 (50)	133 (42)	84 (62)	44 (69)
No	258 (50)	187 (58)	51 (38)	20 (31)
CHAQ Ϯ	0 (0.0–0.63)	0 (0.0–0.38)	0.38 (0.0–1.09)	0.13 (0.0–0.88)
CMAS Strength	50 (44–52)	51 (45–52)	49 (45–52)	48 (37–52)
Pain Score	1 (0–3)	0 (0–2)	2 (0–5)	0 (0–2)
QoL Score ^a^				
Excellent	129 (23)	109 (32)	5 (3)	15 (21)
Good or Very Good	395 (71)	217 (64)	129 (88)	49 (70)
Poor	31 (6)	12 (4)	13 (9)	6 (9)

^a^ Significant univariate association. Discordance defined as at least a 2-point difference in gVAS

Ϯ Data presented as median (interquartile range)

In this JDM cohort, 60 patients (11%) had ongoing weakness described as moderate or severe, 37% (*n* = 122) had a CMAS of < 48, 52% (*n* = 289) had a rash, 42% (*n* = 227) had ongoing nailfold capillaroscopy abnormalities, 10% (*n* = 53) had calcinosis, and 18% (*n* = 97) had abnormal muscle enzymes. Half of patients were receiving oral steroids (*n* = 261), while 14% were receiving IV pulse steroids (*n* = 69; Table 1).

### Comparison of global activity assessment score discordance

Overall, 61% (*n* = 344) of PF and MD gVAS scores were concordant (within 2 points of each other on 10-point VAS scale), 26% (*n* = 149) were discordant with PF rating of gVAS ≥2 points above (worse than) MD, and 12% (*n* = 70) were discordant with MD rating of gVAS worse than PF gVAS. Of the discordant scores (39%; *n* = 219), 68% (n = 149) of PF rated their disease activity as worse than the MD, while 32% (n = 70) of MD rated higher disease activity compared with PF (Table 1). The factors found to be significantly associated with discordance in each of the groups, as well as those with no discordance, are described in Table 2.

**Table 2:** Factors associated with discordance (≥2point difference, with higher score indicating poorer functioning/more severe disease) in gVAS scores between PF and MD (all *p* < 0.01), and those with no effect on discordance

Patient/Family gVAS score higher	Physician gVAS score higher	No effect on discordance
Poorer CHAQ scores	Muscle enzyme abnormalities	Current age/age of onset
Poorer Quality of Life scores	Proximal muscle weakness	Disease duration
	Presence of rash/calcinosis	Gender
	Nailfold capillaroscopy changes	Race/ethnicity
	Joint involvement	Income level
	Current use of steroids	CMAS score
		Gastrointestinal/Cardiac involvement
		Pain score

CHAQ = Childhood Health Assessment Questionnaire; CMAS = Childhood Myositis Assessment Scale

When PT VAS was ≥2 points above MD (indicating poorer functioning/more severe disease), these patients had significantly worse CHAQ scores and more frequently reported poor quality of life (*p* < 0.01). When MD gVAS was ≥2 points above PT gVAS, these patients had more frequent muscle enzyme abnormalities, worse proximal muscle weakness, rash, nail fold changes, calcinosis, higher percentage of joint involvement, and current IV pulse steroid treatment (all *p* < 0.01). Other medications, including biologic medications and oral or subcutaneous methotrexate, were not significantly associated with discordance (*p* = 0.55, 0.08 and 0.07, respectively). Demographic factors, including current age, age of onset, disease duration, gender, race/ethnicity, and income level, were also not associated with discordance in the VAS scores between patients/families and physicians (Table 2).

### Multivariable analysis of factors associated with discordance

Multivariable logistic regression analysis of discordance in global activity assessment, where PF gVAS was rated as ≥2 points higher than MD gVAS, included independent predictors of calcinosis (OR 2.1, 95% CI: 1.2–4.2, *p* = 0.043), CHAQ> 0.125 (OR 2.0, 95% CI: 1.2–3.0, *p* = 0.004) and lower quality of life scores (good/very good vs excellent, OR 12.5, 95% CI: 4.5–25, *p* < 0.001; poor vs. excellent, OR 17.0, 95% CI: 4.6–32, *p* < 0.001).

Multivariable logistic regression of discordance in global activity assessment, where MD gVAS was rated ≥2 points higher than PF gVAS, found several significant independent predictors, including rash (OR 11.0, 95% CI: 3.7–22, *p* < 0.001), calcinosis (OR 3.3, 95% CI: 1.4–8.2, *p* = 0.009), nailfold capillaroscopy changes (OR 2.1, 95% CI: 1.1–4.3, *p* = 0.040), and patients receiving pulse steroids (OR 2.3, 95% CI: 1.3–5.0, *p* = 0.039).

**Fig. 1:**
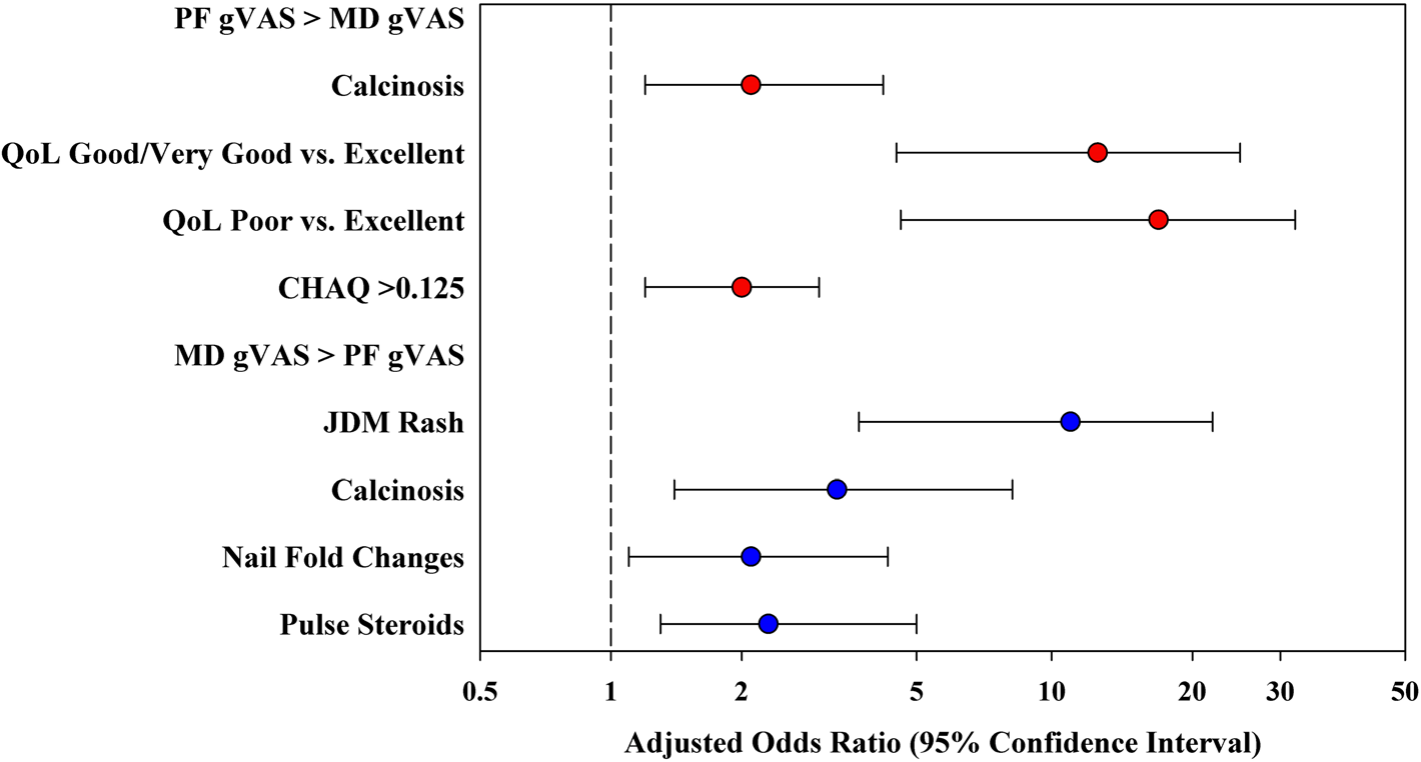
Forest plot illustrating the adjusted odds ratios and 95% confidence intervals for each significant independent predictor of PF and MD discordance in gVAS. Discordance is defined as at least a 2-point difference on the 0–10 VAS scale. The red circles indicate predictors increasing the odds of discordance, where PF > MD. The blue circles denote predictors increasing the odds of discordance, where MD > PF

This multivariable analysis is summarized in a forest plot, with the adjusted odds ratio of discordance and 95% CI for each significant independent predictor (Fig. 1).

## Discussion

There is a rapidly growing focus in healthcare regarding the importance of patient-centered care, with the goal of improving care that is most relevant to patients and families. This focus is especially important in chronic conditions, including juvenile dermatomyositis.

In this analysis of the JDM CLR, we found that in about 60% of cases, patient/parent and physician global assessments of disease activity were similar based on the concordance of their reported global activity scores. However, in approximately 40% of cases, there was significant discordance between the PF and MD reported gVAS scores. We also found that patients/families rated themselves as doing worse compared to their treating physicians two times more often than they rated themselves as doing better (26% vs. 12%), supporting our previous findings that patient/family perspectives vary from health care professionals in JDM [12].

The rate of discordance in this study highlights some limitations in this subjective measure of global disease activity. Discordance between patient/family and physician global activity assessment can lead to difficulty assessing the effectiveness of treatment, particularly in chronic conditions like JDM. In addition, significant discordance in global activity assessment is likely to be associated with lower patient/family satisfaction and decreased adherence to the recommended treatment regimen [13]. While it is possible that some of this discordance could be related to differences in interpretation of the rating scale by patients/families and physicians, the wording of the question to the groups was identical and these measures have undergone validation testing [4]. It is therefore important to understand factors that contribute to discordance, as this may help us to identify better approaches to better capture patient/family perspectives of disease burden and direct the development of better PROs for JDM in the future.

Our exploratory univariate analysis suggested some correlations of interest: poorer CHAQ and Quality of Life score are associated with worse PF gVAS, while elevated muscle enzymes, proximal muscle weakness, rash, arthritis and steroid use is associated with worse MD gVAS. However our multivariable regression analysis gives us some more useful insight into what may drive clinician and patient/family gVAS ratings, as we found certain independent predictors of discordance. Our findings suggest that clinicians may use clinical features, such as rash, nailfold capillaroscopy changes, calcinosis, and medications to inform their global assessment of disease, whereas patients/families may place a greater emphasis on PROs such as global assessment of disease on decreased function (based on CHAQ scores) and quality of life. It is possible that other patient-centered factors, such as fatigue and mental health, could also contribute to patient/family assessments of disease. Pain was not found to be a significant factor associated with discordance in our patient population, but overall pain scores were low in our cohort and pain may not have been a common enough symptom to identify discordance.

Interestingly, in multivariable analysis calcinosis was an independent factor that influenced discordance on gVAS in both directions. Though calcinosis is considered a measure of disease damage rather than activity according to IMACS, there is debate in clinical practice and the literature with regard to whether calcinosis represents disease damage and/or disease activity and it is a common practice to treat calcinosis with increasing immunosuppression [14–16]. This result highlights the important impact of calcinosis on the reported and perceived JDM disease activity by physicians as well as patients, and further evaluation into the factors driving this as an independent predictor of discordance in both directions would be an interesting topic for further study. This result also emphasizes the need to monitor and treat this important clinical manifestation of disease. Though we do not know the type and extent of calcinosis of patients from the CLR, we suspect that patients with calcinosis and worse PF gVAS scores likely had lesions that contributed to functional limitations, pain and concerns regarding physical appearance, in addition to other factors not measured in the registry.

The importance of capturing the patient/family perspective of disease activity in JDM cannot be minimized. In addition to the importance of including patient/family perspectives, PROs may be able to help better *inform* clinicians of disease activity in myositis, as it has in other conditions. For example in adult myositis, patients with reduced health related quality of life scores had lower muscle strength [17]. Dynamic repetitive muscle function has also been found to correlate with patient-reported physical function [18].

Fortunately, international organizations conducting research in JDM, including IMACS and PRINTO, were prescient to include specific PROs as part of their disease core set measures, such as patient global assessment; however, there remains more work to be done in this field. As our work suggests, the extent to which these PROs capture the aspects of the disease that are important to patients/families has not been well studied, and these tools were originally developed with limited patient input. In addition, currently existing PROs were not developed specifically for inflammatory myositis. Outcome Measures in Rheumatology (OMERACT), an international initiative interested in outcome measures in rheumatology, has a myositis working group to develop, examine and validate PROs in adult myositis [19]. To improve the outcomes of our patients with JDM, it will be important to extend this work to patients with pediatric myositis in the future.

As with any study, there are limitations to our findings. Overall, patients enrolled in the CLR trended toward milder disease and the median disease duration in this cohort was short, which may limit the generalizability of the findings. In addition, since this was a cross sectional cohort, assessment of these measures and determination of concordance at specific time points (e.g., at diagnosis, remission, flares, etc.) was not possible. Differences in disease duration or severity could be expected to impact responses from patients and families; however, in our cohort there was no significant difference in disease duration between the physician and patient/family groups who had concordant gVAS or discordant gVAS in either direction. We also do not know if patients or parents filled out the PF gVAS. This could complicate interpretation of the results, since previous studies have shown differences in responses to patient reported outcome metrics when assessed by the patient or a family member/care giver [20, 21]. It would have been interesting to assess differences in patient compared to physician scores, as well as patient compared to parent scores, and this would be an interesting topic for future study. Furthermore, it is possible that the gVAS score could be interpreted differently between different physicians; however, training on the use of these tools was available through CARRA and associated research groups, and previous studies have assessed inter-rater reliability of physician gVAS in inflammatory disorders with good results [22].

We were limited in the number of outcomes we could assess, and the CLR collected a limited number of PROs. Some PROs of particular importance, such as measures of fatigue, anxiety and depression, were not collected, which could potentially also impact gVAS score results. The current updated version of the CARRA Registry is more comprehensive and includes additional PROs, which should be incorporated into future studies.

## Conclusions

In summary, we found a nearly 40% rate of discordance between patient/family and physician global activity assessment scores in patients with JDM enrolled to the CLR. Overall, worse patient/family scores were associated with worse PROs, while worse MD scores were associated with poorer objective measures of disease activity. Our findings suggest that PF and MD gVAS may often measure different facets of JDM disease activity and burden. Our work underscores the need to develop alternative relevant and valid patient-focused outcome measures that can be integrated into our overall assessment of patients with JDM, for use not only in clinical trials, but also in clinical decision-making and routine care of patients with JDM to improve future outcomes from this disease.

## Data Availability

The datasets analyzed during the current study are available from the corresponding author on reasonable request.

## Abbreviations

CARRA: Childhood Arthritis and Rheumatology Research Alliance
CHAQ: Childhood Health Assessment Questionnaire
CMAS: Childhood Myositis Assessment Scale
gVAS: Global Disease Activity Score
IMACS: International Myositis Assessment and Clinical Studies Group
JDM: Juvenile Dermatomyositis
MD: Physician
OMERACT: Outcome Measures in Rheumatology
PF: Patient or Family
PRINTO: Pediatric International Trials Organisation
PROs: Patient Reported Outcome Measures
VAS: Visual Analog Scale
